# Light-absorbing organic carbon from prescribed and laboratory biomass burning and gasoline vehicle emissions

**DOI:** 10.1038/s41598-017-06981-8

**Published:** 2017-08-04

**Authors:** Mingjie Xie, Michael D. Hays, Amara L. Holder

**Affiliations:** 1 Oak Ridge Institute for Science and Education (ORISE), 109 T.W. Alexander Drive, Research Triangle Park, NC 27711 USA; 2 U.S. Environmental Protection Agency, Office of Research and Development, National Risk Management Research Laboratory, 109 T.W. Alexander Drive, Research Triangle Park, NC 27711 USA

## Abstract

Light-absorbing organic carbon (OC), also termed brown carbon (BrC), from laboratory-based biomass burning (BB) has been studied intensively to understand the contribution of BB to radiative forcing. However, relatively few measurements have been conducted on field-based BB and even fewer measurements have examined BrC from anthropogenic combustion sources like motor vehicle emissions. In this work, the light absorption of methanol-extractable OC from prescribed and laboratory BB and gasoline vehicle emissions was examined using spectrophotometry. The light absorption of methanol extracts showed a strong wavelength dependence for both BB and gasoline vehicle emissions. The mass absorption coefficients at 365 nm (MAC_365_, m^2^ g^−1^C) – used as a measurement proxy for BrC – were significantly correlated (*p* < 0.05) to the elemental carbon (EC)/OC ratios when examined by each BB fuel type. No significant correlation was observed when pooling fuels, indicating that both burn conditions and fuel types may impact BB BrC characteristics. The average MAC_365_ of gasoline vehicle emission samples is 0.62 ± 0.76 m^2^ g^−1^C, which is similar in magnitude to the BB samples (1.27 ± 0.76 m^2^ g^−1^C). These results suggest that in addition to BB, gasoline vehicle emissions may also be an important BrC source in urban areas.

## Introduction

Carbonaceous aerosols are ubiquitous in the atmosphere and can directly affect Earth’s climate by absorbing and scattering incoming solar radiation^1–3^. Specifically, the black carbon (BC) component of carbonaceous aerosols, or soot, absorbs strongly across the spectral range (from ultraviolet [UV] to the infrared [IR]) showing a weak dependence on wavelength (λ)^4–6^. In direct contrast, the organic carbon (OC) component of aerosol is commonly treated as purely light scattering or “white”^7, 8^. The optical properties of these aerosols appear in many climate models that show scattering due to OC causes a cooling effect that offsets the warming effect due to BC^9, 10^. However, growing evidence suggests that certain chemical components of OC can absorb in the near UV and at shorter visible wavelengths impacting radiative forcing^11–14^. This component is often referred to as brown carbon (BrC). These light-absorbing BrC components may also influence aerosol photochemistry (e.g., photolysis) and health effects^15^.

Both field^16^ and laboratory measurements^14, 17–19^ have confirmed that biomass burning (BB) is an important primary source of BrC, which is also clearly observed in BB-impacted atmospheres^20–23^. There is also evidence of secondary BrC formation. For example, laboratory chamber studies indicate BrC formation following the photooxidation of volatile organic compounds (VOCs) emitted from biogenic (e.g., isoprene), BB (e.g., *m*-cresol), and motor vehicle (e.g., toluene) sources^17, 24–27^. However, the optical properties of OC emitted from other sources, particularly from fossil fuel combustion, are largely unstudied.

Based on existing studies of BrC from laboratory simulated BB, large variability in the spectral dependence associated with the chemical variability of BrC constituents has been observed^28–31^. In addition, the water-insoluble fraction of BrC has much stronger light absorption efficiency than the water-soluble fraction of BrC, and the light-absorbing efficiency of BrC depends largely on burn conditions (e.g., temperature)^14, 15, 18, 32^. However, chemical and optical information about the BrC emitted from prescribed or controlled burning is scant^16, 33^. Prescribed burning is a less intensive fire technique used in forest and agricultural land management, or for land restoration objectives. Prescribed agricultural burns prepare fields for planting, stimulate plant growth and yields, and control pests, whereas prescribed forest burning is used to abate aggressive wildfire and promote ecological succession and sustainability^16^. Despite the benefits, prescribed burning emits pollutants (e.g., particulate matter (PM), OC, and VOCs) that can have serious regional air quality impact^34^. For example, Tian *et al*.^35^ simulated the impact of BB emissions on PM_2.5_ in Georgia using a chemical transport model, which ascribed more than 50% of the regional PM_2.5_ to prescribed BB during January and March, 2002.

Motor vehicles are also a primary source of PM_2.5_ emissions to urban atmospheres. Lee *et al*.^36^ estimated PM_2.5_ source contributions to the southeastern United States using positive matrix factorization and chemical mass balance models, showing that motor vehicles contributed 17–25% of PM_2.5_ in urban areas, 6–13% greater than wood burning. However, the association between BrC and motor vehicle aerosol emissions is less certain than for BB. Kirchstetter and Novakov^11^ suggest that low-temperature incomplete combustion similar to what can occur during BB produces light-absorbing (organic) aerosol with much stronger spectral dependence than higher-temperature combustion processes like diesel combustion. Interestingly, Liu *et al*.^37^ investigated BrC based on water and methanol extracts of aerosols collected at urban, rural and near-road sites, finding that the near-road mass absorption efficiencies of water extracts are higher (>40%) than at the urban site. The derived absorbing component of the complex refractive index (*k*) of near-road aerosol was used to represent gasoline sources by Lu *et al*.^38^, which is subject to large uncertainty. However, direct measurements of BrC from primary vehicle emissions are still lacking.

This study attempts to address limitations in understanding BrC as it relates to primary source combustion emissions. In that vein, UV-Vis spectrometry was applied to measure the light-absorbing properties of OC in methanol extracts of prescribed and laboratory BB and gasoline vehicle aerosol emissions. The BB tests were conducted using a variety of fuels and fire conditions. We hypothesized that both the BB conditions and fuel type would impact the OC absorptivity. The gasoline vehicle emissions were sampled during different seasons (winter and summer) while also examining vehicle class (truck and car) and model year variables.

## Methods

### Sampling of prescribed burn

Table 1 provides the field location, fuels, and trial population for the prescribed burns. Kentucky blue grass residues (*Poa prantensisi L*., “KBG”), wheat stubble (*Triticum aestivum L*., “Wheat”), and chemically fallowed wheat stubble (“Wheat + Herbicide”) were burned in field in the northwestern United States (Nez Perce, ID and Walla Walla, WA). A grass plot consisting of various species of grasses, forbs, and turkey oak (*Quercus laevis*) and a forest plot of primarily long leaf pine (*Pinus palustris*) were burned at a forest field in the southeastern United States (Eglin Air Force Base, FL). Further description of the prescribed burns is given in supplementary information and Table S1, and Holder *et al*.^39^ exhaustively describe the forest prescribed burning. The sampling methods and instrumentation applied here were identical to those applied previously^16, 33, 39^. Briefly, both ground and aerial (aerostat) sampling with identical instrumentation packages were deployed during the prescribed burns. One instrumentation package, including continuous measurements of CO_2_ (LICOR-820, LICOR Bioscience), BC (AE51, Aethlabs) and particle size distribution (DustTrak DRX 8533, TSI), and batch sampling of PM_2.5_ (Impactor, SKC) etc., was attached to a helium filled, tethered aerostat (4.3 m in diameter) as the aerial sampling platform; a second instrumentation package was mounted on an all-terrain vehicle as the ground sampling platform. For the duration of each burn, PM_2.5_ was sampled at 10 L/min on Teflon^TM^ and quartz filters (QF, diameter 43 mm, Pall) positioned downstream of a PM_2.5_ cyclone (URG). Multiple filters (up to three) were sequentially collected for selected burns to avoid overloading. At each field location, a background sample was obtained upwind of the burn capturing ambient air throughout the burn duration.

**Table 1:** Description of the location, fuel species and sample numbers for prescribed burns, and sample numbers for corresponding laboratory simulations at the open burn test facility (OBTF).

Location	Fuel type	Field sample No.	OBTF sample No.
**Agriculture Field**			
Nez Perce, ID	Kentucky Bluegrass (“KBG”)	6^a^	3
Nez Perce, ID	Wheat stubble (“Wheat”)	2^a^	3
Walla Walla, WA	Chemically fallowed wheat stubble (“Wheat + Herbicide”)	6^a^	3
**Forest Field** ^b^			
Eglin Air Force Base, FL	Grass/forb/shrub/wood debris (“Forest burn”)	4^c^	9
Eglin Air Force Base, FL	Grass/forb/shrub (“Grass burn”)	2^a^	0

^a^Contain equal numbers of ground and aerostat samples.

^b^Aurell *et al*.^33^; Holder *et al*.^39^

^c^Contain 3 ground and 1 aerostat samples.

### Laboratory fire simulations

With the exception of the “grass burn” at Eglin Air Force Base, FL, a corresponding laboratory fire simulation was conducted in an attempt to mimic prescribed burns. Fire simulations were conducted at the U.S. EPA (RTP, NC) Open Burn Test Facility (OBTF). Biomass fuel collection and fire simulation methods are described in detail elsewhere^33, 39^. Briefly, biomass fuels — gathered at the prescribed burn sites — were divided and burned in batches on an aluminum foil-coated steel pan in a 70 m^3^ enclosure. For consistency, the same instrumentation package used for field sampling was used for OBTF sampling. Background air samples were collected post-burn inside the OBTF.

### Light-duty vehicle emissions

PM_2.5_ samples collected from gasoline vehicle exhaust were selected from the Kansas City Light-Duty Vehicle Emissions Study (KCVES) filter archive. Vehicle data, emissions testing protocols, and sampling details are given elsewhere^40–43^. In summary, the KCVES study separated passenger cars and light-duty trucks into four model year groups representing different technologies: carburetors (pre-1981), early fuel injectors (1981–1990), phase in Tier-1 standards (1991–1995) and National Low Emission Vehicles (1996–2005)^43^. Exhaust emissions from 496 vehicles recruited from the Kansas City metropolitan area were measured in two rounds: round 1 summer (261 vehicles), and round 2 winter (235 vehicles). Vehicles were tested on a portable chassis dynamometer in a warehouse at ambient temperature using the LA92 Unified Driving Cycle. The LA92 cycle is 15.7 km and consists of three operating phases, including “cold start” (phase 1), “hot running” (phase 2) and “hot start” (phase 3). Vehicle exhaust was cooled and diluted and drawn through a PM_2.5_ cyclone, followed by 47 mm Teflon^TM^ and QF filters. PM_2.5_ samples were collected for each of the three phases of the LA92 cycle. In the present study, PM_2.5_ QF samples were selected from both rounds of emissions testing. Supplementary Table S2 provides vehicle selections, including make and model, model year, and sampling temperatures. Dilution tunnel blanks were also examined and treated as a background check. Finally, all sampled air volumes (1.73 ± 0.12 m^3^) and dilution ratios were virtually identical throughout emissions testing.

### Analytical chemical procedures

Multiple studies have shown that methanol extracts aerosol OC at higher efficiencies than water, and that a large fraction of light absorption in the near-UV and visible ranges is ascribed to water-insoluble OC^23, 32, 37^. Hence, methanol was used for sample extractions. For prescribed and laboratory BB samples, a QF filter punch (1.5 cm^2^) was extracted with 5 mL methanol (HPLC grade) in a tightly closed amber vial, sonicated for 15 min, and then filtered (National Scientific Company, 30 mm diameter. ×0.2 μm pore size, polytetrafluoroethylene (PTFE)) using a glass syringe. The light absorption of filtered extracts was measured with a UV-Vis spectrometer at λ = 200–900 nm and a resolution of 0.2 nm (V660, Jasco Incorporated, Easton MD). The wavelength accuracy and repeatability were checked monthly to ensure the quality of the data being collected. The wavelength accuracy was less than ±0.3 nm; the wavelength repeatability was less than ±0.05 nm. A reference cuvette containing methanol was used to eliminate the impact of solvent absorption. The UV-Vis absorption of background air samples was negligible (greater than 1 order of magnitude lower) compared to prescribed burn samples but used for correction nonetheless. This study focused on λ = 300–550 nm, where most of the BrC absorption has been observed^32^.

All BB QF samples were analyzed for OC and elemental carbon (EC) content using a thermal-optical instrument (Sunset Laboratory, Portland, OR) and modified National Institute of Occupational Safety and Health (NIOSH), Method 5040^44^. Instrument blanks and calibration check standards (sucrose solution) were run at the beginning of each day to ensure valid measurements. Only trace concentrations of OC were observed in background air samples, accounting for less than 1% of the average OC concentration in BB samples, and were used for correction. The amount of OC extracted was calculated as the difference between OC on the un-extracted QF and OC in the air-dried residual QF following extraction. The OC extraction efficiency was calculated as the ratio of extracted OC to OC on the un-extracted filter multiplied by 100%.

Analytical procedures for gasoline vehicle emissions samples were virtually identical to the BB samples, the only difference being that the three QFs corresponding to each phase of the LA92 cycle were composited and analyzed. In other words, one punch of each of the three QF filters in every selected run was combined (three punches in total) and extracted for spectroscopic measurement. The OC and EC were also measured (three punches together) prior to and after filter extraction. The background air in the testing warehouse was impacted by residual vehicle emissions, so the light absorption, OC and EC content of dilution tunnel blank samples were provided separately and not subtracted for correction. The extraction efficiency of OC was also calculated.

### Data analysis

The light absorption measured by the UV-Vis spectrometer is expressed as:

1 $${A}_{\lambda }=\,\mathrm{log}(\frac{{I}_{0}}{I})$$

where *A*_*λ*_ is the light absorbance at a given wavelength (λ); *I*_*0*_ and *I* are the intensity of the incident and transmitted light, respectively.

The *A*_*λ*_ value of each sample extract is converted to a light absorption coefficient (Abs_*λ*_, Mm^−1^) by ref. 20

2 $$Ab{s}_{\lambda }=({A}_{\lambda }-{A}_{700})\times \frac{{v}_{l}}{{v}_{a}\times L}\,\mathrm{ln}(10)$$

where *A*_700_ is referenced to account for systematic baseline drift^45^, *V*_*l*_ (m^3^) is the volume of methanol (5 mL) used for extraction, *V*_*a*_ (m^3^) is the volume of the sampled air represented by the extracted filter punches, and *L* (0.01 m) is the optical path length of the quartz cuvette in the UV-vis spectrometer. The bulk mass absorption coefficient (MAC_*λ*_, m^2^ g^−1^C) could be used to describe the absorption efficiency of extracted OC and the value at 365 nm was typically used as a measure of BrC^20^. The MAC_*λ*_ was calculated as^45^:

3 $$MA{C}_{\lambda }=\frac{Ab{s}_{\lambda }}{{C}_{OC}}$$

where *C*_OC_ is the mass concentration of extracted OC in PM (μg m^−3^). Here, the solution MAC_*λ*_ is different from the widely known term “mass absorption cross-section” (*α*_abs_), which is attributed to carbonaceous components in particles suspended in the air. The *α*_abs_ is empirically parameterized as^12^:

4 $${\alpha }_{{\rm{a}}{\rm{b}}{\rm{s}}}=K\times {\lambda }^{-{{\AA }}_{{\rm{a}}{\rm{b}}{\rm{s}}}}$$

where *K* is a fitting parameter including aerosol mass concentrations, and *Å*_abs_ is the absorption Ångstrӧm exponent, a measure of the λ dependence of aerosol light absorption. In this work, the *Å*_abs_ of methanol extract is determined by the linear regression of log_10_(Abs_λ_) vs. log_10_(λ) over the λ range of 300 and 550 nm, which is used to represent the characteristics of BrC (*Å*_abs_ much bigger than 1). All OC and EC measurements and calculations of EC/OC ratio, extraction efficiency, MAC_365_ and *Å*_abs_ for BB and gasoline vehicle emissions samples are provided in supplementary Tables S1 and S2, respectively.

### Data Availability

Data used in the writing of this manuscript can be obtained upon request to Amara Holder (holder.amara@epa.gov).

## Results and Discussion

### Analysis of BB samples

Compared with field-based prescribed burns, Table 2 shows that the fire simulations produce systematically higher EC/OC ratios in the filter PM irrespective of biomass fuel species. In tandem with the higher Modified Combustion Efficiency (MCE) — a measure of a fire’s flaming versus smoldering combustion — observed for the fire simulations (MCE > 0.95^39^). One explanation is that our laboratory simulations were run in an enclosure under very controlled conditions which produced a relatively stable emissions stream and were only able to partially mimic field conditions. These results imply that the simulations produce flaming combustion synonymous with higher burning temperatures. Combining the field measurements, which exhibited lower MCEs and lower EC/OC ratios, with the laboratory simulations results in a range of combustion conditions for the same fuel types. EC/OC ratios determined for the aerostat filter samples are generally higher than ground-level determinations. Presumably, the ground-level samples depict higher smoldering combustion contributions or higher dilution aloft partly vaporizes the semivolatile OC^39^. The extraction efficiency of OC from the BB emissions was consistently 90% or greater, similar to the results of Chen and Bond^32^. The sampling site and fuel and fire type variables show slight if any effect on the extraction efficiency. Table 2 also provides the light absorption properties of extractable OC. For ease of analysis, the bulk mass absorption coefficient of extracted OC at 365 nm (MAC_365_) was used to measure BrC, because the light absorption at this wavelength is representative and has been successfully used to study BrC in past studies^20, 27, 37, 45^. Mean values (EC/OC, extraction efficiency, MAC_365_ and *Å*_abs_) are reported with standard deviations. For tests with *N* < 3, only the mean value is provided.

**Table 2:** EC/OC ratios, OC extraction efficiency and light absorption of organic aerosol from prescribed and laboratory burns.

Fuel type	Sampling type	EC/OC	Extraction efficiency (%)	MAC_365_ (m^2^ g^−1^C)	*Å*_abs_ (300–550 nm)
KBG	Aerostat	0.036 ± 0.011	93.4 ± 0.84	1.38 ± 0.033	7.03 ± 0.068
	Ground	0.032 ± 0.015	94.7 ± 1.91	1.32 ± 0.17	7.12 ± 0.15
	OBTF	0.17 ± 0.091	94.5 ± 2.01	1.80 ± 0.15	6.25 ± 0.26
Wheat	Aerostat	0.084	90.1	1.19	7.82
	Ground	0.018	90.8	1.06	8.11
	OBTF	0.33 ± 0.18	94.5 ± 2.97	1.28 ± 0.12	5.28 ± 0.96
Wheat + Herbicide	Aerostat	0.046 ± 0.019	90.8 ± 3.59	1.05 ± 0.059	7.77 ± 0.51
	Ground	0.016 ± 0.0081	95.1 ± 1.18	1.00 ± 0.076	7.93 ± 0.64
	OBTF	0.13 ± 0.022	91.5 ± 3.17	2.09 ± 0.12	5.83 ± 0.69
Forest burn	Aerostat	0.041	96.5	1.10	7.08
	Ground	0.026 ± 0.0095	97.5 ± 1.13	1.04 ± 0.084	7.37 ± 0.078
	OBTF	0.21 ± 0.16	97.0 ± 1.87	1.13 ± 0.15	7.36 ± 0.59
Grass burn	Aerostat	0.086	95.1	0.90	6.43
	Ground	0.089	95.3	0.97	6.92

**Figure 1:**
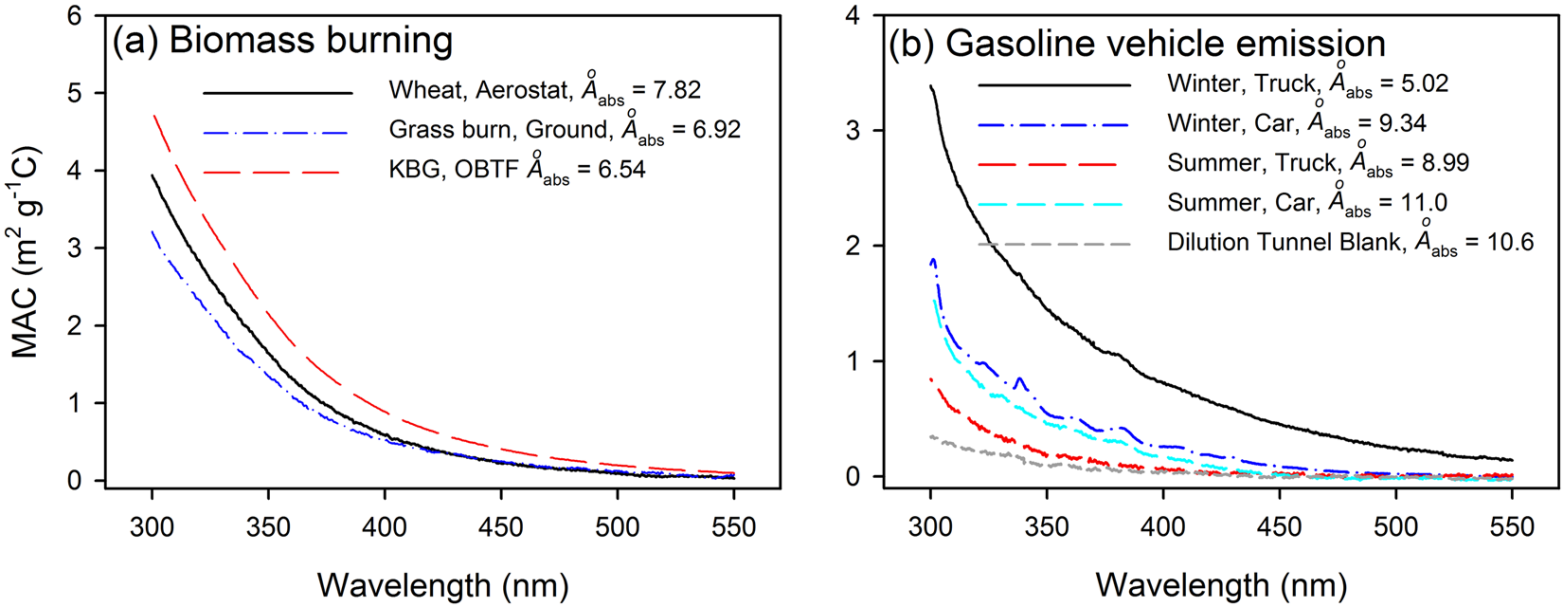
Representative MAC spectra for PM_2.5_ samples from (**a**) Biomass burning and (**b**) gasoline vehicle emissions.

Figure 1a shows characteristic MAC spectra within the wavelength (λ) range of 300–550 nm. The spectra exhibit a strong λ dependence and *Å*_abs_ > 2. Although the absorption is greater over the λ < 350 nm region, absorption at visible wavelengths (>400 nm) is taken as evidence of BrC^15^. The average MAC_365_ and *Å*_abs_ values determined for aerostat, ground and OBTF test samples for each fuel type are listed in Table 2. The methanol extracts of PM from the laboratory fire simulations show relatively high MAC_365_ and low Å_*abs*_ values, suggesting that the higher temperature flaming combustion that dominates the fire simulations preferentially generates OC with strong light absorption. In this work, the MAC_365_ ranged from 0.90 to 2.22 m^2^ g^−1^C across all samples (including aerostat, ground and OBTF); the *Å*_abs_ range is 4.43–8.67 with an average of 7.01 ± 0.90, and the correlations between log_10_(Abs_λ_) and log_10_(λ) are greater than 0.98 (*p* < 0.01). The average MAC_365_ (1.27 ± 0.34 m^2^ g^−1^C) measured in this study is comparable to the methanol extracts for ambient aerosols from the LA basin (MAC_365_ 1.58 m^2^ g^−1^C, Å_*abs*_ 4.82)^45^ and Beijing (MAC_365_ 1.45 ± 0.26 m^2^ g^−1^C, Å_*abs*_ 7.10 ± 0.45)^23^, but greater than those from three sampling sites in Georgia (MAC_365_, 0.27–0.41 m^2^ g^−1^C, Å_*abs*_ 4.02–5.89)^37^. The absorption of methanol-extractable OC measured in the Beijing study was strongly correlated with levoglucosan – a biomass burning tracer, indicating an influence from BB^23^. However, in the LA basin study, BrC was attributed mainly to anthropogenic emissions and associated formation of secondary organic aerosol^45^. The low absorption of aerosol extracts in Georgia was attributed primarily to biogenic secondary emissions^46^. The average Å_*abs*_ (7.01 ± 0.90) in this work is comparable to the extractable OC from burning of corn stalks (7.7)^47^ and wood (6.9–7.8)^32^.

### Light absorption and EC/OC ratio

**Figure 2:**
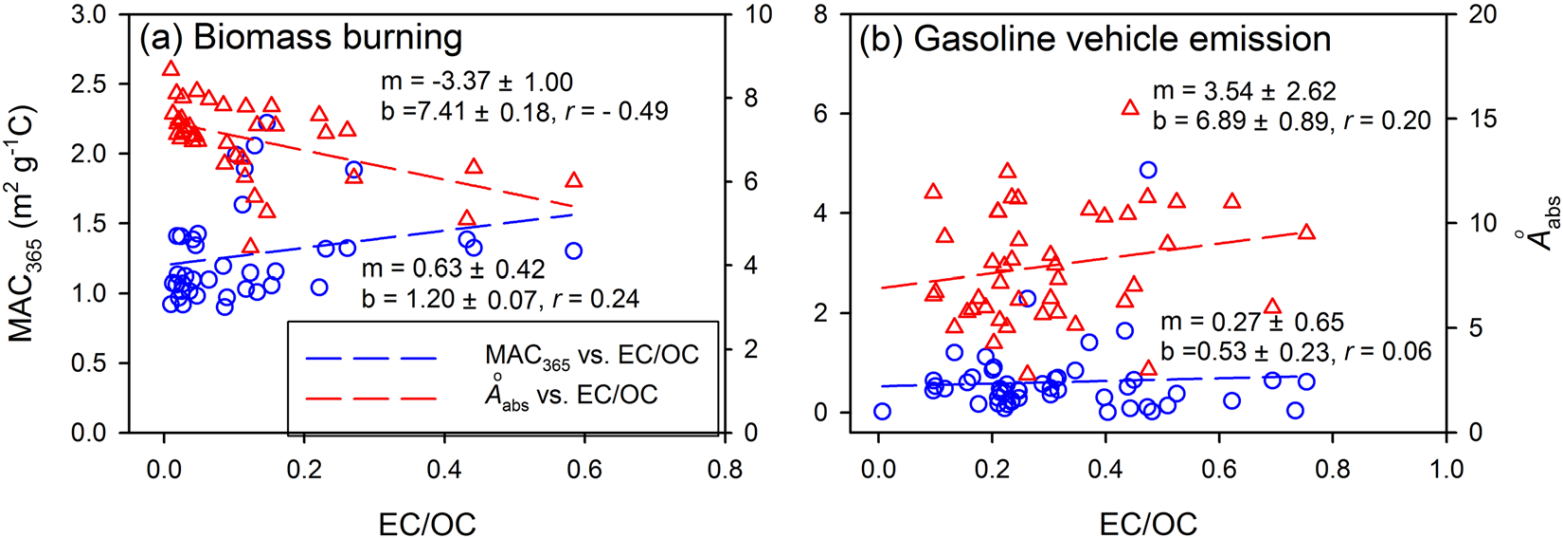
Linear regressions of MAC_365_ vs. EC/OC, and *Å*_abs_ vs. EC/OC for (**a**) prescribed and laboratory biomass burning and (**b**) gasoline vehicle emissions. In each plot, m and b represent regression slope and intercept, respectively, with one standard error.

Instead of MCE, which has shown only moderate correlation with optical properties^48^, the EC/OC ratio is used here as an indicator of fire conditions. Recent studies comparing MCE and EC/OC have shown that EC/OC is key to understanding aerosol optical properties^18, 38^. Figure 2a shows the MAC_365_ vs. EC/OC and *Å*_abs_ vs. EC/OC relationships for all the BB samples (regardless of fuel types and sampling method). The data clearly show that the light absorption of OC from BB is dependent on burn conditions as measured by EC/OC, consistent with previous studies^14, 18^. However, the scatter and low MAC_365_ and EC/OC correlation (*r* = 0.24, *p* > 0.05) suggest that something other than fire conditions may influence the light-absorbing properties of OC from BB.

**Figure 3:**
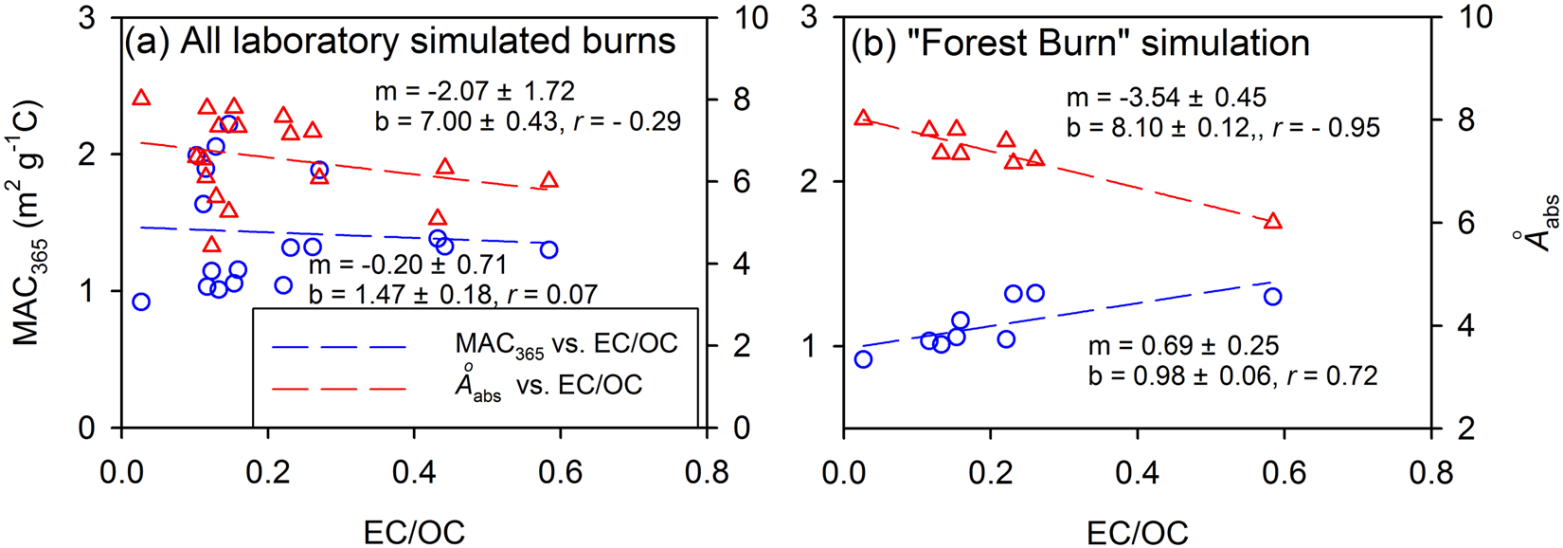
Linear regressions of MAC_365_ vs. EC/OC, and *Å*_abs_ vs. EC/OC for (**a**) all laboratory simulated burns (OBTF) and (**b**) laboratory simulations for “Forest burn”. In each plot, m and b represent regression slope and intercept, respectively, with one standard error.

Previous laboratory studies of BrC from BB have observed that optical properties depend on burning conditions but not on fuel type^14, 38^. However, these studies^14, 38^ are limited in that there were few replicates per fuel type and the replicates tended to reflect similar burning conditions. Thus, a limited range of EC/OC values was observed per fuel type, and the *Å*_abs_ and MAC_365_ relationships could therefore not be adequately characterized by fuel type. To compare with Saleh *et al*.^14^, we pooled all OBTF samples together (Fig. 3a) and compared to only the forest fuels (Fig. 3b), which had the greatest sample population (*N* = 9) of the laboratory burn simulations and the widest EC/OC range. When the OBTF results are pooled, the MAC_365_ vs. EC/OC (*r* = 0.07, *p* > 0.05) and *Å*_abs_ vs. EC/OC (*r* = −0.29, *p* > 0.05) linear correlations are not statistically significant. When limited to just the forest fuels, the regressions for MAC_365_ and *Å*_abs_ with EC/OC become statistically significant (*p* < 0.05; Fig. 3b). The uncertainties in EC/OC ratio, MAC_365_ and *Å*_abs_ were estimated by replicate analysis of select filter samples to determine if measurement uncertainty impacts the MAC_365_ and *Å*_abs_ dependence on EC/OC. While the potential variability intrinsic to the combustion system was not addressed. Details of the uncertainty analysis are provided in the Supplementary Information and Tables S3 and S4. The uncertainty was around 5% for the EC/OC ratio and less than 5% for MAC_365_ and *Å*_abs_. Therefore, the linear relationship and overall trends observed for MAC_365_ vs. EC/OC and *Å*_abs_ vs. EC/OC in Fig. 3b are unaffected by measurement uncertainty, further confirming the importance of burning conditions and biomass fuel type on the light absorption of OC from BB.

In addition, Figure S1 shows MAC_365_ vs. EC/OC and *Å*_abs_ vs. EC/OC by fuel type for all sampling methods (aerostat, ground and OBTF) fit to a linear regression (“Grass burn” data are removed because sample number *N* = 2). All correlations are significant (*p* < 0.05) except for *Å*_abs_ vs. EC/OC (*r* = −0.49, *p* > 0.05, Figure S1c) for the “Wheat” fires. The weak correlation observed for “wheat” fires may be due to the limited sample population (*N* = 5), although the MAC_365_ and EC/OC correlation for “Wheat” is significant (*r* = 0.95, 0.01 < *p* < 0.05). In Figure S1, the field data points have little variation in MAC_365_ and *Å*_abs_, suggesting similar burn conditions during filed burns. Except “Grass burn”, the aerostat samples had consistently higher average EC/OC ratio and MAC_365_, and lower *Å*_abs_ than ground samples (Table 2), which might also indicate the dependence of light absorption on burn condition. Among the four biomass fuels in Figure S1, MAC_365_ and *Å*_abs_ of the “Wheat + herbicide” burns show the highest sensitivity to the EC/OC ratio (slope, 9.87 and −19.5), while the “Wheat” and “Forest burn” tests are the least sensitive (slope < 1), suggesting that fuel type influences the optical properties of OC from BB. However, how biomass fuel type affects the light absorption of OC from BB is not clear and warrants further study. In this work, due to the small sample populations for laboratory simulated burns using “KBG”, “Wheat” and “Wheat + Herbicide”, and the relatively narrow EC/OC ratio and MAC_365_ and *Å*_abs_ ranges for the field measurements, future studies are needed to verify the relationships of MAC_365_ vs. EC/OC and *Å*_abs_ vs. EC/OC for specific biomass fuel types.

Saleh *et al*.^14^ evaluated the OC light absorption on a direct particle measurement basis, which may in part be influenced by BC absorption. In our study, the OC component is extracted and isolated from EC, thus there is no EC influence or lensing effect (enhancement of EC absorption by OC coatings). These different approaches may have impacted the relationship between fuel type and BrC, which may partly explain the differences between this study and that of Saleh *et al*.^14^. Additionally, Saleh *et al*.^14^ analyzed emissions from a different fuel species set compared with the current study. To date, few studies have investigated the influence of burning conditions and fuel types on BrC from BB emissions^14, 18, 32^, which is necessary to predict the impact of BB aerosols on radiative forcing^12^.

### Analysis of gasoline vehicle emissions samples

To date, a majority of studies propose BB as a major BrC source, which has led to relatively limited testing of anthropogenic sources for BrC content. An objective of the present study is to examine the potential BrC contribution due to petroleum-powered vehicles. Supplementary Table S2 provides pertinent vehicle and emissions test information by study trial as well as the OC and EC concentrations, EC/OC ratios, OC extraction efficiency, MAC_365_ and *Å*_abs_. The OC and EC concentrations are reported as μg m^−3^ and potentially reflect the emission strength because the total sampling and dilution volumes are similar for all tests. Due to sample availability, the pre-1981 and 1981–1990 vehicle groups are combined and compared with the 1991–1995 and 1996–2005 vehicle groups. Emissions from all tested vehicles were combined for data trend analysis and data visualization.

The OC and EC concentrations (μg m^−3^), EC/OC ratios, and OC extraction efficiency results by vehicle group and season are given in Supplementary Figure S2. As expected, OC and EC emissions increase in winter and with vehicle age. Schauer *et al*.^49^ found that motor vehicles emitted substantially more carbonaceous particle matter at low temperatures (~0 °C) than at regular temperatures (~24 °C). Similar trends with season and vehicle age were observed previously for PM emissions^42, 43^. EC/OC ratios are greater in the summer, contrasting with the extraction efficiency of OC, perhaps owing to increased volatilization of SVOCs and faster catalyst and engine warm-up times. EC/OC ratios and OC extraction efficiency correlate negatively (*r* = −0.52, *p* < 0.01) for all vehicle test data. Presumably, the EC in PM strongly adsorbs OC, in turn reducing the extraction efficiency.

**Figure 4:**
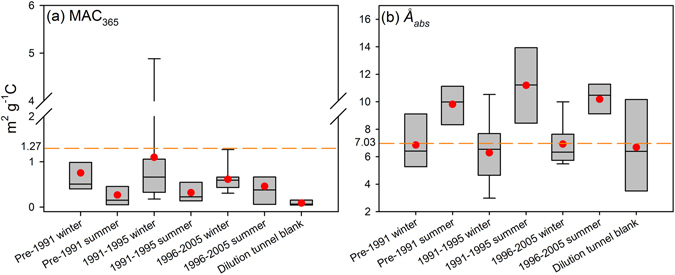
Seasonal box plots for (**a**) MAC_365_ and (**b**) *Å*_abs_ for different model year vehicles emissions. The boxes depict the median (dark line in the box), inner quartile range (gray box), 10^th^ and 90^th^ percentiles (whiskers) and the average (red circle). The orange dash lines represent the average MAC_365_ and *Å*_abs_ for biomass burning samples.

In Fig. 1b, typical MAC spectra over the 300–550 nm λ range are shown for gasoline vehicle emissions. Similar to BB, light absorption is observed in both the UV and short visible regions, and spectra exhibit strong wavelength dependence (*Å*_abs_ > 2). Figure 4 shows the MAC_365_ and *Å*_abs_ values by season for the vehicle groups. The median and average MAC_365_ values were higher in winter (median 0.51–0.66 m^2^ g^−1^C, average 0.61 ± 0.34–1.10 ± 0.66 m^2^ g^−1^C) than in summer (0.15–0.38 m^2^ g^−1^C, 0.27 ± 0.30–0.46 ± 0.46 m^2^ g^−1^C), while *Å*_abs_ values exhibited an opposite seasonal variation (winter average 6.29 ± 2.25–6.93 ± 1.53, summer average 9.81 ± 1.50–10.18 ± 1.27). Unlike the OC and EC emissions, the median and average MAC_365_ did not show a consistent trend across vehicle model year. These results suggest that gasoline vehicles could generate stronger light-absorbing OC emissions under colder conditions, although the season had less of an effect on the light-absorbing properties of OC from vehicles with newer model years (1996 to 2005).

Compared with the BB, the MAC_365_ values for gasoline vehicle emissions are generally lower (average 0.62 m^2^ g^−1^C) and more variable (range, 0.016–4.88 m^2^ g^−1^C). However, the average or median MAC_365_ values of these sources are of similar magnitude. Hence, gasoline vehicle emissions may represent a substantial contribution to BrC in urban regions affected by vehicle emissions. Interestingly, the EC/OC ratio may not be an adequate proxy for understanding the light absorption of methanol extractable OC in gasoline vehicle emissions. Neither the MAC_365_ nor the *Å*_abs_ correlate to EC/OC (*r* = 0.06, *p* > 0.05 and *r* = 0.20, *p* > 0.05; Fig. 2b). This observation is valid even when isolating samples by season (winter and summer), vehicle type (truck and car), or model year. Therefore, the BrC from gasoline vehicles may be compositionally different than BrC from BB. The extraction efficiency of OC from gasoline vehicle emissions (75.9 ± 9.42%) is lower than the extraction efficiency of OC from BB (>90%), and the light absorption of the residual OC is uncertain. Chen and Bond^32^ found that higher BB temperatures can generate more light-absorbing OC and suggest that macromolecules containing both conjugated aromatic rings and functional groups are responsible for the light absorption. Di Lorenzo and Young^50^ compared the contributions of high- and low-molecular weight compounds to light absorption using aged BB aerosols and observed large molecular weight (>1000 Da) components as the dominant contributors. Thermodenuder measurements performed to characterize light-absorbing OC in BB aerosols as a function of volatility also demonstrated that extremely low volatility OC contributed most to light absorption^14^. The non-extracted OC fraction in gasoline vehicle emissions is likely hydrophobic or high molecular weight compounds with conjugated double-bonded carbon structures (e.g., PAHs). Their light absorption properties require further study.

### Imaginary part of the complex refractive index

In this study, the *k* value of extractable organic matter — another measure of BrC — from BB and gasoline vehicle emissions was calculated based on the spectroscopic data measured in this study. The calculation method and resulting *k* values are given in the Supplementary Information and Table S5, respectively. The median *k* values of BB samples at wavelengths of 365, 405 and 550 nm are 0.026, 0.014 and 0.0020, respectively, and of the same magnitude as those *k* values estimated by Lack *et al*.^51^ (404 nm, 0.009) and Li *et al*.^47^ (400 nm, 0.041; 550 nm, 0.005) but 5–10 times lower than the values from Saleh *et al*.^14^ (550 nm, ~0.01–0.03). Li *et al*.^47^ calculated the *k* value using the same method as this study (solvent extracts based); Lack *et al*.^51^ and Saleh *et al*.^14^ performed optical closure with Mie theory calculations to retrieve effective *k* values. The optical closure method has large uncertainties since the real particle morphology may greatly deviate from the idealized spherical Mie model. The discrepancies between these studies in *k* estimation might be caused by the difference in both method and BB (biomass fuel and burn conditions). The median *k* values of gasoline vehicle emissions at 365, 405 and 550 nm are 0.013, 0.0086 and 0.0015 in winter, respectively, and approximately two times the values in summer. Therefore, treating organic aerosol as non-absorbing particles would underestimate the radiative effect of organic aerosols, especially in urban areas where motor vehicle emissions are a substantial fraction of the aerosol.

This study measured the light absorption of methanol-extractable OC derived from BB and gasoline vehicle emissions, which exhibited strong wavelength dependence with Åabs values much higher than 2. The OC generated during BB under high temperature or flaming combustion shows strong light absorption; the biomass fuel type may also play a role in the light-absorbing properties of OC generated from BB. However, how biomass fuel type affects the light absorption of OC from BB is uncertain and merits further study. Gasoline vehicles tend to emit stronger light-absorbing OC in winter than in summer. Compared to BB, the light absorption of OC from gasoline vehicle emissions was of the same magnitude but weaker, suggesting the importance of gasoline vehicle emissions as a BrC source in urban regions. Non-extractable OC accounted for a substantial part (~25%) of the total OC from gasoline vehicle emissions, and further study to measure its potential light-absorbing properties is warranted.

## Electronic supplementary material

Supplementary Information
