# Supplementary material for: Light-absorbing organic carbon from prescribed and laboratory biomass burning and gasoline vehicle emissions
Source: Sci Rep. 2017 Aug 4;7:7318. doi: 10.1038/s41598-017-06981-8 (PMC5544734; doi:10.1038/s41598-017-06981-8)
Supplement: Supplementary file 1 — Supplementary Information [file 41598_2017_6981_MOESM1_ESM.pdf]

Supplementary information for

"Light-absorbing organic carbon from prescribed and laboratory biomass burning and gasoline vehicle emissions"

Mingjie Xie, Michael D. Hays, Amara L. Holder*

U.S. Environmental Protection Agency, Office of Research and Development, National Risk Management Research Laboratory, 109 T.W. Alexander Drive, Research Triangle Park, NC 27711, USA

Correspondence to: Amara L. Holder;

E-mail: holder.amara@epa.gov;

Tel: +1 919 541 4635;

Fax: +1 919 541 0554

Mailing address: 109 T.W. Alexander Dr. RTP, NC, 27709, USA

Pages Number: 16

Table Number: 5

Figure Number: 2

Supplementary methods

1. Prescribed burns in the agriculture and forest field

A number of prescribed agriculture burns were conducted in the northwestern United States (Nez Perce, ID and Walla Walla, WA). The fires were carried out during August 19 – 25, 2013. Kentucky Blue Grass (*Poa prantensis L.*, KBG) and Winter Wheat (*Triticum aestivum L.)* were burned in fields of Nez Perce, ID, while the fuels burned in fields of Walla Walla, WA, were considered "chemically fallowed winter wheat" where the wheat had been treated with an herbicide (2,4-dichlorophenoxyacetic acid). A series of prescribed forest fires were conducted in the southeastern United State (Eglin Air Force Base, FL). A grass and shrub (mixed biomass of grass forb and shrub) plot and a forest (mixed biomass of grass, forb, shrub and wood debris) plot were burned in November, 2012, respectively. Each field consisted of a single fuel and was burned sequentially to ensure that samples were taken from emissions of only one fuel type. One sample was collected per field throughout the duration of the burn. All prescribed burns were ignited by a drip torch with a mixture of gasoline and diesel fuels. The detailed sample information was given in Table S1.

2. Uncertainty in EC/OC ratio for biomass burning (BB) sample

The uncertainty in EC/OC ratio for each BB sample is mainly derived from the measurement of OC and EC in filter samples. The uncertainties in OC and EC measurements in this study were estimated by duplicate analysis of four selected samples, including two background air samples and two biomass burning samples. As shown in Table S3, the average relative percent difference (RPD) of OC, EC and EC/OC are 0.43%,

0.98% and 5.14%, respectively, which were used as the relative uncertainty in OC, EC and EC/OC ratio.

3. Uncertainty of MAC_{365} and $Å_{abs}$ for biomass burning sample

The uncertainties in MAC_{365} and $Å_{abs}$ were estimated by replicate analysis of four selected BB samples. Due to the limit of particle loading area on filter (~ 11.5 cm^2), three small filter punches (0.385 cm^2) were cut from each selected BB filter sample and extracted separately, followed by UV-Vis analysis as replicate measurements. Following the data analysis method introduced in the manuscript, the MAC_{365} and $Å_{abs}$ values for the three replicate measurements of each filter were calculated and listed in Table S4, along with the original measurement data used in the manuscript. In Table S4, MAC_{365} and $Å_{abs}$ had small variability for replicate analysis of each sample, as reflected by the relative standard deviation (RSD, <5%). As such, the uncertainties of MAC_{365} and $Å_{abs}$ due to measurement should be lower than 5%.

4. Calculation of imaginary refractive index

The parameterization of optical properties of organic aerosol (OA) is necessary in modeling their climate effects. The complex refractive index ($m = n - ik$) is the most commonly used parameter to determine particles' optical properties, where the real (n) and the imaginary (k) parts corresponding to the scattering and absorbing properties, respectively. The imaginary part of the refractive index (k) of OA from prescribed burns and gasoline vehicle emissions ould be calculated from their spectroscopic data using equation S1[1,2]:

$$k = \frac{\rho \times \lambda \times \mathrm{Abs}_{\lambda}}{4 \times \pi \times \mathrm{OM}} = \frac{\rho \times \lambda \times \mathrm{MAC}_{\lambda}}{4 \times \pi} \times \frac{1}{\mathrm{OM/OC}} \qquad \text{(S1)}$$

where Abs_{λ} ($\mathrm{Mm^{-1}}$) is the light absorption coefficient, MAC_{λ} ($\mathrm{m^2\ g^{-1}C}$) is the bulk mass absorption coefficient, OM ($\mu\mathrm{g\ m^{-3}}$) is the concentration of extracted organic matter, OC ($\mu\mathrm{g\ m^{-3}}$) is the concentration of extracted organic carbon, and ρ ($\mathrm{g\ cm^{-3}}$) is the density of organic aerosols. In this work, the density of organic aerosols is assigned as 1.2 $\mathrm{g\ cm^{-3}}$ based on the estimate from[3], and an OM/OC ratio of 1.5 was selected based on some measurement studies [4-6]. The *k* values for BB and gasoline vehicle emissions samples are given in Supplementary Table S5.

Supplementary Table S1. Sample information for the prescribed burns in agriculture and forest fields, and their corresponding laboratory simulations in open burn test facilities (OBTF).

Date	Fuel	Sample type	Sample volume (m^3)	OC (mg m^{-3})	EC (mg m^{-3})	EC/OC	Extraction efficiency (%)	MAC_{365} (m^2 $g^{-1}C$)	$Å_{abs}$
Agriculture field burn									
8/19/2013	KBG	Aerostat	0.20	4.81	0.20	0.041	92.7	1.38	6.96
8/20/2013	KBG	Aerostat	0.21	6.16	0.15	0.024	94.3	1.41	7.02
8/20/2013	KBG	Aerostat	0.18	1.48	0.070	0.045	93.1	1.34	7.09
8/19/2013	KBG	Ground	0.37	3.01	0.14	0.048	96.8	1.42	6.97
8/20/2013	KBG	Ground	0.15	0.81	0.024	0.03	93.0	1.12	7.27
8/20/2013	KBG	Ground	0.27	5.58	0.10	0.018	94.4	1.41	7.13
11/13/2013	KBG	OBTF	0.12	4.54	0.51	0.11	95.7	1.63	6.54
11/13/2013	KBG	OBTF	0.18	3.92	0.45	0.12	95.8	1.89	6.11
11/13/2013	KBG	OBTF	0.19	1.97	0.53	0.27	92.2	1.88	6.09
8/20/2013	Wheat	Aerostat	0.057	2.74	0.23	0.084	90.1	1.19	7.82
8/20/2013	Wheat	Ground	0.081	1.82	0.033	0.018	90.8	1.06	8.11
11/14/2013	Wheat	OBTF	0.13	1.74	0.75	0.43	93.5	1.38	5.09
11/14/2013	Wheat	OBTF	0.20	1.65	0.73	0.44	92.1	1.32	6.33
11/14/2013	Wheat	OBTF	0.22	3.71	0.46	0.12	97.8	1.15	4.43
8/24/2013	Wheat + Herbicide	Aerostat	0.38	0.94	0.025	0.026	93.9	1.07	7.19
8/24/2013	Wheat + Herbicide	Aerostat	0.20	1.61	0.10	0.064	91.5	1.10	7.96
8/25/2013	Wheat + Herbicide	Aerostat	0.16	1.21	0.056	0.047	86.8	0.98	8.16
8/24/2013	Wheat + Herbicide	Ground	0.47	1.97	0.049	0.025	95.8	1.02	7.50
8/24/2013	Wheat + Herbicide	Ground	0.26	2.51	0.032	0.013	95.7	1.07	7.62
8/25/2013	Wheat + Herbicide	Ground	0.15	1.57	0.016	0.010	93.7	0.92	8.67
11/14/2013	Wheat + Herbicide	OBTF	0.13	5.85	0.60	0.10	94.1	1.99	6.60
11/14/2013	Wheat + Herbicide	OBTF	0.23	1.44	0.21	0.15	92.4	2.22	5.26
11/14/2013	Wheat + Herbicide	OBTF	0.18	1.45	0.19	0.13	88.0	2.06	5.62

Supplementary Table S1. Continue

Date	Fuel	Sample type	Sample volume (m^3)	OC (mg m^{-3})	EC (mg m^{-3})	EC/OC	Extraction efficiency	MAC_{365}	$Å_{abs}$
				Forest field burn					
11/11/2012	Forest Burn	Aerostat	1.84	0.59	0.024	0.041	96.5	1.10	7.08
11/11/2012	Forest Burn	Ground	1.39	1.51	0.055	0.036	98.0	1.01	7.31
11/11/2012	Forest Burn	Ground	0.23	6.24	0.13	0.021	98.3	0.97	7.47
11/11/2012	Forest Burn	Ground	0.15	4.26	0.082	0.019	96.2	1.13	7.38
11/16/2012	Forest burn	OBTF	0.45	1.90	0.22	0.12	98.8	1.03	7.79
11/16/2012	Forest burn	OBTF	0.13	3.82	0.58	0.15	99.1	1.06	7.80
11/16/2012	Forest burn	OBTF	0.14	1.56	0.91	0.58	98.6	1.30	6.00
11/16/2012	Forest burn	OBTF	0.26	14.1	0.38	0.027	98.6	0.92	8.01
11/16/2012	Forest burn	OBTF	0.26	2.76	0.44	0.16	95.6	1.16	7.33
11/16/2012	Forest burn	OBTF	0.19	6.14	0.81	0.13	96.1	1.01	7.34
11/16/2012	Forest burn	OBTF	0.21	4.96	1.09	0.22	97.0	1.04	7.58
11/16/2012	Forest burn	OBTF	0.21	2.77	0.64	0.23	95.9	1.32	7.15
11/16/2012	Forest burn	OBTF	0.23	3.46	0.90	0.26	93.6	1.32	7.22
11/10/2012	Grass burn	Aerostat	0.36	0.31	0.026	0.086	95.1	0.90	6.43
11/10/2012	Grass burn	Ground	0.42	0.27	0.024	0.089	95.3	0.97	6.92

Supplementary Table Table S2. Information of selected runs of vehicle emission tests

Run No.	Winter										
	t (°C)	Vehicle type	Make	Model	Year	OC[a]	EC[a]	EC/OC	Extraction efficiency (%)	MAC_{365}[b]	$Å_{abs}$
84703	6.5	Truck	Chevrolet	Blazer	1987	308	54.3	0.18	79.5	0.18	6.41
84720	6.5	Truck	Chevrolet	1500 PU	1989	371	83.7	0.23	73.2	0.57	5.04
84665	12.7	Truck	Toyota	4X4 PU	1989	316	59.7	0.19	59.6	1.12	5.98
84683	7.5	Truck	Ford	Ranger PU	1990	172	52.1	0.30	65.7	0.37	6.41
84519	-1.3	Truck	Dodge	Caravan	1992	514	178	0.35	76.4	0.84	5.15
84517	-2.4	Truck	Dodge	Caravan	1992	584	118	0.20	81.1	0.91	4.27
84620	11.0	Truck	Ford	Ranger PU	1992	183	24.4	0.13	79.3	1.21	5.02
84768	13.4	Truck	Ford	F150 PU	1995	81.2	7.86	0.10	73.7	0.66	6.54
84473	0.7	Truck	Ford	Explorer	1996	183	127	0.69	64.9	0.65	5.96
84564	4.5	Truck	Dodge	Caravan	1998	182	44.7	0.25	75.6	0.30	6.33
84537	4.9	Truck	Jeep	Cherokee	2000	122	30.1	0.25	85.7	0.45	9.16
84558	6.4	Truck	Chevrolet	S-10 PU	2001	576	250	0.43	53.7	1.65	6.24
84563	3.6	Truck	Ford	Ranger 4X4 PU	2003	64.3	10.0	0.16	70.8	0.64	5.76
84708	7.8	Truck	Dodge	Caravan	2003	123	55.5	0.45	55.7	0.67	7.00
84562	0.7	Truck	Dodge	Dakota PU	2004	104	21.8	0.21	68.0	0.32	10.5
84588	9.0	Car	Buick	Regal	1978	673	78.3	0.12	81.4	0.48	9.34
84659	13.0	Car	Chrysler	Le Baron	1988	371	163	0.44	70.3	0.52	10.4
84682	6.1	Car	Toyota	Camry	1990	393	103	0.26	75.2	2.29	2.76
84669	11.7	Car	Dodge	Spirit	1990	678	205	0.30	89.5	0.50	8.46
84723	12.6	Car	Chevrolet	Cavalier	1991	352	35.2	0.10	86.8	0.53	6.71
84575	3.9	Car	Chevrolet	Lumina	1994	171	81.3	0.48	53.5	4.88	2.99
84714	6.2	Car	Saturn	SW	1994	370	77.9	0.21	71.0	0.18	10.5
84452	8.0	Car	Ford	Taurus	1995	201	47.1	0.23	80.2	0.25	8.23
84406	-6.2	Car	Toyota	Corolla	1995	1310	281	0.21	84.2	0.41	7.14
84629	3.8	Car	Acura	TL2.5	1996	121	38.2	0.32	69.9	0.47	7.32
84416	-2.1	Car	Honda	Civic	1998	88.7	18.9	0.21	70.7	0.50	5.37
84515	-2.1	Car	Dodge	Stratus	1999	171	54.1	0.32	81.1	0.70	5.72
84438	4.6	Car	Buick	Century	2001	76.1	16.9	0.22	72.4	0.42	7.95
84520	-2.9	Car	Ford	Taurus	2001	92.1	26.6	0.29	74.1	0.59	5.64
84514	-2.8	Car	Chrysler	Concorde	2002	118	48.2	0.41	75.9	0.62	7.06

[a] μg m^{-3}; [b] m^2 $g^{-1}C$; [c] NAs of MAE_{365} represent measurements lower than the median of laboratory blanks, and NAs of $Å_{abs}$ are due to the light absorption from 300 to 550 nm mostly lower than the median of laboratory blanks.NA values are not included for statistics.

Supplementary Table S2.Continue

Run No.	Summer										
	t (°C)	Vehicle type	Make	Model	Year	OC	EC	EC/OC	Extraction efficiency (%)	MAC_{365}	$\mathring{A}_{abs}$
84248	26.3	Truck	Ford	Ranger	1987	187	95.0	0.51	66.5	0.15	8.99
84354	26.6	Truck	Ford	F150	1989	306	61.3	0.20	88.7	0.86	8.11
84256	27.6	Truck	Chevy	S10 P/U	1989	189	118	0.62	68.2	0.24	11.0
84373	22.0	Truck	Chevrolet	Astrovan	1990	354	78.8	0.22	78.0	0.087	NA
84094	25.6	Truck	Ford	Explorer	1993	63.9	17.1	0.27	74.8	NA[c]	NA
84302	26.2	Truck	Ford	F150 P/U	1993	110	48.9	0.44	67.3	0.10	15.5
84268	24.8	Truck	Ford	F150 P/U	1994	167	39.2	0.23	78.9	0.23	11.2
84242	26.2	Truck	Plymouth	Voyager	1997	428	159	0.37	62.8	1.43	10.6
84329	28.5	Truck	Jeep	Wrangler	1997	166	15.9	0.10	86.6	0.45	11.4
84055	35.5	Truck	Jeep	Cherokee	1998	103	48.6	0.47	64.8	0.13	11.2
84298	23.3	Truck	Dodge	Durango	1999	347	108	0.31	72.7	0.68	8.00
84096	25.9	Truck	Isuzu	Rodeo	1999	97.7	67.6	0.69	72.7	NA	NA
84376	19.5	Truck	Honda	Odyssey	2000	125	49.6	0.40	83.4	0.31	10.3
84150	20.1	Truck	Honda	Odyssey	2000	98.6	47.5	0.48	75.7	0.037	NA
84213	24.3	Car	Olds	Regency 98	1985	105	29.9	0.28	81.3	NA	NA
84221	27.9	Car	Ford	LTD Crown Victoria	1985	262	64.3	0.25	85.4	0.46	11.2
84107	30.0	Car	Buick	LeSabre	1989	106	43.6	0.41	83.0	NA	NA
84208	24.3	Car	Lincoln	Towncar	1989	1643	10.9	0.0067	94.1	0.016	NA
84141	25.5	Car	Honda	Accord	1990	156	115	0.73	68.6	0.052	NA
84228	30.4	Car	Pontiac	Grand Am	1992	236	124	0.52	73.0	0.38	11.0
84347	25.5	Car	Toyota	Corolla	1995	251	41.4	0.16	85.8	0.71	5.88
84182	24.8	Car	Ford	Thunderbird	1995	279	.6.99	0.025	90.1	NA	NA
84274	23.6	Car	Ford	Aspire	1995	158	35.8	0.23	83.0	0.18	12.4
84369	17.3	Car	Volvo	850 Wagon	1996	158	119	0.75	77.3	0.63	9.50
84227	29.9	Car	Buick	Century	1997	48.8	1.46	0.030	78.8	NA	NA
84280	26.0	Car	Ford	Escort	1999	60.6	2.75	0.045	91.4	NA	NA
84272	22.4	Car	Honda	Accord	2001	66.7	26.9	0.40	85.2	0.036	NA
84224	28.4	Car	Ford	Taurus	2002	52.5	1.68	0.032	86.2	NA	NA

Supplementary Table S2. Continue

Run No.	Dilution tunnel blanks										
	Temp (°C)	Vehicle Type	Make	Model	Year	OC	EC	EC/OC	Extraction efficiency (%)	MAC_{365}	$Å_{abs}$
84410	-8.0	/	/	/	/	30.3	0.52	0.017	89.3	0.044	8.77
84691	12.7	/	/	/	/	31.8	1.36	0.043	89.0	0.075	10.6
84549	10.9	/	/	/	/	29.6	0.72	0.024	93.3	0.059	4.02
84555	6.5	/	/	/	/	22.8	0	0	87.4	NA	NA
84776	16.4	/	/	/	/	25.7	1.39	0.054	88.6	0.19	3.33
84106	34.0	/	/	/	/	23.5	0.59	0.025	93.2	NA	NA

Supplementary Table S3. Duplicate analysis of OC and EC in selected sample filters.

Sampling date	Sample Type	Fuel	OC (mg cm^{-2})[a]	RPD of OC (%)[b]	EC (mg cm^{-2})[a]	RPD of EC (%)	OC/EC	RPD of EC/OC (%)
11/14/2013	Agriculture Field Background Air	/	5.90	0.50	0.14	0.00	0.024	2.01
	Duplicate	/	6.02		0.14		0.023	
11/19/2012	OBTF Background Air	/	4.80	0.36	0.19	0.00	0.040	1.45
	Duplicate	/	4.87		0.19		0.039	
11/11/2012	Ground	Forest Burn	57.26	0.61	1.10	0.45	0.019	4.24
	Duplicate	Forest Burn	55.88		1.12		0.020	
08/25/2013	Aerostat	Wheat + herbicide	16.80	0.25	0.77	3.47	0.046	12.9
	Duplicate	Wheat + herbicide	16.63		0.67		0.040	

[a] values directly from Sunset ECOC instrument; [b] relative percentage difference (%) between the measurements of two punches from the same filter, RPD = 2 × abs (X1-X2)/(X1+X2) × 100%;

Supplementary Table S4. Replicate analysis of MAC and $Å_{abs}$ for selected biomass burning filters.

Sampling date	Sample Type	Fuel	MAC_{365} (m^2 g^{-1})	RSD[b] of MAC_{365} (%)	$Å_{abs}$	RSD of $Å_{abs}$ (%)
8/20/2013	Aerostat[a]	KBG	1.41	2.12	7.02	3.51
	replicate1	KBG	1.44		6.83	
	replicate2	KBG	1.48		7.35	
	replicate3	KBG	1.42		7.33	
11/11/2012	Aerostat[a]	Forest Burn	1.10	4.79	7.08	3.16
	replicate1	Forest Burn	1.16		6.88	
	replicate2	Forest Burn	1.12		7.10	
	replicate3	Forest Burn	1.04		7.42	
11/11/2012	Ground[a]	Forest Burn	1.01	4.32	7.31	1.95
	replicate1	Forest Burn	1.09		7.02	
	replicate2	Forest Burn	1.00		7.30	
	replicate3	Forest Burn	0.99		7.29	
11/11/2012	Ground[a]	Forest Burn	0.97	1.27	7.47	3.02
	replicate1	Forest Burn	0.94		7.67	
	replicate2	Forest Burn	0.95		7.36	
	replicate3	Forest Burn	0.97		7.88	

[a] measurement data used in the manuscript; [b] relative standard deviation = standard deviation (including all 4 measurements) /average × 100%.

Supplementary Table S5. Imaginary refractive index (*k*) of organic aerosols from BB and gasoline vehicle emissions.

λ	Median	Average	stdev[a]	min	max	% not detected[b]
Prescribed and laboratory burns (N = 38)						
365	0.026	0.030	0.0079	0.021	0.052	0
405	0.014	0.016	0.0055	0.0096	0.033	0
550	0.0020	0.0026	0.0018	0.00020	0.0085	0
Gasoline vehicle emissions						
Winter (N=30)						
365	0.013	0.018	0.021	0.0041	0.11	0
405	0.0086	0.013	0.018	0.0022	0.098	0
550	0.0015	0.0045	0.0097	0.00021	0.044	23.3
Summer (N = 28)						
365	0.0055	0.0083	0.0083	0.00037	0.033	28.6
405	0.0034	0.0050	0.0050	0.000039	0.018	42.9
550	0.00057	0.00066	0.00058	0.000072	0.0020	67.9
Dilution tunnel blanks (N=6)						
365	0.0016	0.0021	0.0015	0.0010	0.0043	33.3
405	0.0011	0.0015	0.0013	0.00053	0.0034	33.3
550	0.0017	0.0017	0.00063	0.0013	0.0021	66.7

[a] standard deviation
[b] percentage of samples with no absorption

Figure Captions

Supplementary Figure S1. Linear regressions of MAC_{365} vs. EC/OC, and $\AA_{abs}$ vs. EC/OC for specific biomass fuels, (a) Forest burn, (b) KBG, (c) Wheat and (d) Wheat + Herbicide. Open symbols represent field burns, and filled ones represent laboratory simulated burns (OBTF); m and b represent regression slope and intercept, respectively, with one standard error.

Supplementary Figure S2. Seasonal box plots for (a) OC concentrations, (b) EC concentrations, (c) EC/OC ratios and (d) extraction efficiency of OC for different model year vehicles emissions. The boxes depict the median (dark line in the box), inner quartile range (gray box), 10th and 90th percentiles (whiskers) and the average (red circle).

Figure S1

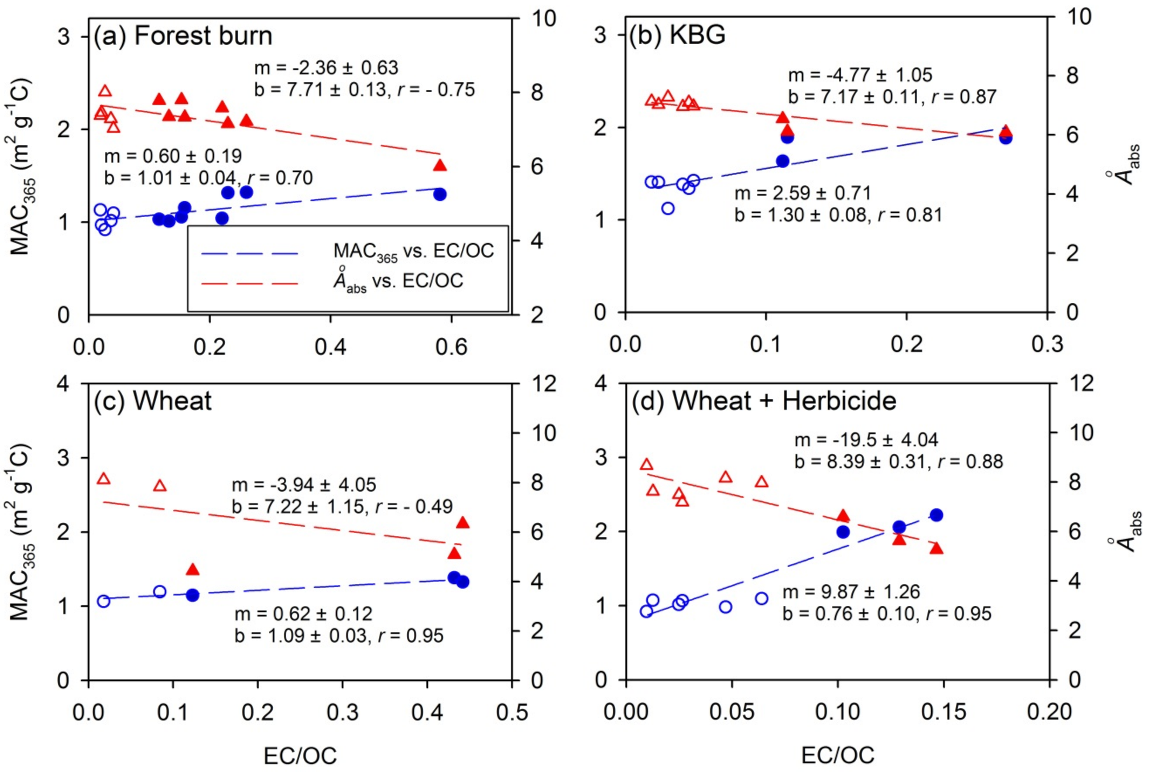

(a) Forest burn
m = -2.36 ± 0.63
b = 7.71 ± 0.13, r = - 0.75
m = 0.60 ± 0.19
b = 1.01 ± 0.04, r = 0.70
MAC365 vs. EC/OC
Åabs vs. EC/OC
MAC365 (m2 g-1C)
(b) KBG
m = -4.77 ± 1.05
b = 7.17 ± 0.11, r = 0.87
m = 2.59 ± 0.71
b = 1.30 ± 0.08, r = 0.81
Åabs
(c) Wheat
m = -3.94 ± 4.05
b = 7.22 ± 1.15, r = - 0.49
m = 0.62 ± 0.12
b = 1.09 ± 0.03, r = 0.95
(d) Wheat + Herbicide
m = -19.5 ± 4.04
b = 8.39 ± 0.31, r = 0.88
m = 9.87 ± 1.26
b = 0.76 ± 0.10, r = 0.95
EC/OC
EC/OC

Figure S2

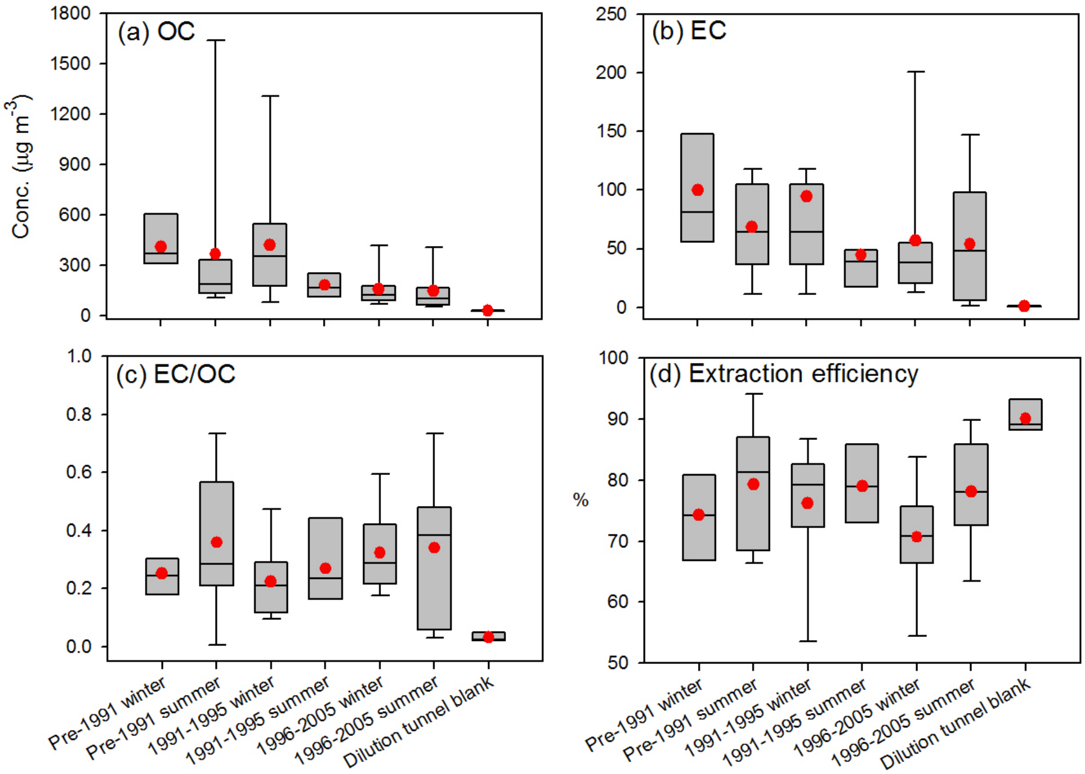

(a) OC
Conc. (µg m-3)
1800
1500
1200
900
600
300
0
(b) EC
250
200
150
100
50
0
(c) EC/OC
1.0
0.8
0.6
0.4
0.2
0.0
(d) Extraction efficiency
%
100
90
80
70
60
50
Pre-1991 winter
Pre-1991 summer
1991-1995 winter
1991-1995 summer
1996-2005 winter
1996-2005 summer
Dilution tunnel blank

Supplementary References

1. Lu, Z. *et al.* Light absorption properties and radiative effects of primary organic aerosol emissions. *Environ. Sci. Technol.* **49**, 4868-4877, doi:10.1021/acs.est.5b00211 (2015).
2. Liu, J. *et al.* Optical properties and aging of light-absorbing secondary organic aerosol. *Atmos. Chem. Phys.* **16**, 12815-12827, doi:10.5194/acp-16-12815-2016 (2016).
3. Turpin, B. J. & Lim, H.-J. Species contributions to PM2.5 mass concentrations: Revisiting common assumptions for estimating organic mass. *Aerosol Sci. Tech.* **35**, 602-610, doi:10.1080/02786820119445 (2001).
4. Aiken, A. C. *et al.* O/C and OM/OC ratios of primary, secondary, and ambient organic aerosols with high-resolution time-of-flight aerosol mass spectrometry. *Environ. Sci. Technol.* **42**, 4478-4485, doi:10.1021/es703009q (2008).
5. Brown, S. G., Lee, T., Roberts, P. T. & Collett, J. L. Variations in the OM/OC ratio of urban organic aerosol next to a major roadway. *J. Air Waste Manage.* **63**, 1422-1433, doi:10.1080/10962247.2013.826602 (2013).
6. Xing, L. *et al.* Seasonal and spatial variability of the OM/OC mass ratios and high regional correlation between oxalic acid and zinc in Chinese urban organic aerosols. *Atmos. Chem. Phys.* **13**, 4307-4318, doi:10.5194/acp-13-4307-2013 (2013).
